# Neural correlates of suspiciousness and interactions with anxiety during emotional and neutral word processing

**DOI:** 10.3389/fpsyg.2014.00596

**Published:** 2014-06-27

**Authors:** Joscelyn E. Fisher, Gregory A. Miller, Sarah M. Sass, Rebecca Levin Silton, J. Christopher Edgar, Jennifer L. Stewart, Jing Zhou, Wendy Heller

**Affiliations:** ^1^Department of Psychology, University of Illinois at Urbana-ChampaignChampaign, IL, USA; ^2^Department of Psychiatry, Center for the Study of Traumatic Stress, Uniformed Services University of the Health SciencesBethesda, MD, USA; ^3^Department of Psychology, University of California Los AngelesLos Angeles, CA, USA; ^4^Department of Psychiatry and Biobehavioral Sciences, University of California Los AngelesLos Angeles, CA, USA; ^5^Department of Psychology and Counseling, University of Texas at TylerTyler, TX, USA; ^6^Department of Psychology, Loyola UniversityChicago, IL, USA; ^7^Department of Radiology, The Children’s Hospital of PhiladelphiaPhiladelphia, PA, USA; ^8^Department of Psychiatry, University of California San DiegoSan Diego, CA, USA

**Keywords:** suspiciousness, anxiety, emotional stroop, paranoia, event-related brain potentials

## Abstract

Suspiciousness is usually classified as a symptom of psychosis, but it also occurs in depression and anxiety disorders. Though how suspiciousness overlaps with depression is not obvious, suspiciousness does seem to overlap with anxious apprehension and anxious arousal (e.g., verbal iterative processes and vigilance about environmental threat). However, suspiciousness also has unique characteristics (e.g., concern about harm from others and vigilance about social threat). Given that both anxiety and suspiciousness have been associated with abnormalities in emotion processing, it is unclear whether it is the unique characteristics of suspiciousness or the overlap with anxiety that drive abnormalities in emotion processing. Event-related brain potentials were obtained during an emotion-word Stroop task. Results indicated that suspiciousness interacts with anxious apprehension to modulate initial stimulus perception processes. Suspiciousness is associated with attention to all stimuli regardless of emotion content. In contrast, anxious arousal is associated with a later response to emotion stimuli only. These results suggest that suspiciousness and anxious apprehension share overlapping processes, but suspiciousness alone is associated with a hyperactive early vigilance response. Depression did not interact with suspiciousness to predict response to emotion stimuli. These findings suggest that it may be informative to assess suspiciousness in conjunction with anxiety in order to better understand how these symptoms interact and contribute to dysfunctional emotion processing.

## INTRODUCTION

Anxiety, depression, and psychotic disorders are often comorbid (e.g., Sands and Harrow, 1995; Braga et al., 2004; Baillie and Rapee, 2005). Researchers have increasingly emphasized the importance of determining which symptoms are unique to each disorder or shared with other disorders and how these symptoms interact (e.g., Braga et al., 2004). Suspiciousness (or paranoia in its extreme) is a symptom that involves the exaggerated tendency to believe that other people intend harm, especially to oneself. It is believed to exist on a continuum (Combs et al., 2002) and is associated with anxiety and depression in the general population (Martin and Penn, 2001; Messias and Kirkpatrick, 2001; Ellett et al., 2003; von Gemmingen et al., 2003; Combs and Penn, 2004) and in schizophrenia-spectrum disorders (Kirkpatrick et al., 1996; Freeman and Garety, 1999; Messias et al., 2001; Candido and Romney, 2002; Goodwin et al., 2002; Drake et al., 2004; Spitznagel and Suhr, 2004; Huppert and Smith, 2005). Although rarely investigated, a better understanding of the role of suspiciousness in the context of anxiety and depression could foster improved definition, classification, and treatment of psychological disorders.

Suspiciousness may be a manifestation or consequence of severe anxiety and depression. This possibility is consistent with Foulds and Bedford’s (1975) hierarchical model of psychopathology in which individuals with disorders at higher levels (e.g., psychosis) have all the symptoms of the lower levels (e.g., mood disorders and anxiety). They proposed that comorbidity exists because severe symptoms at higher levels are episodic and therefore do not camouflage lower-level symptoms at all times. The model is also supported by the fact that individuals with one disorder are at increased risk for meeting criteria for another diagnosis, suggesting that the presence of certain symptoms makes more severe symptoms more likely. Since suspiciousness is more often associated with psychosis than with anxiety and depression, it may be a “higher-level” symptom that may develop as a consequence of depression and/or anxiety.

Other models also propose that emotional disorders have common trait characteristics, such as general distress or negative affect (Clark and Watson, 1991; Zinbarg and Barlow, 1996; Brown et al., 1998). As these models are based on assessments of depression and anxiety, and suspiciousness is usually considered characteristic of psychosis, suspiciousness has not been studied in relation to these models. However, given the comorbidity outlined above and evidence that emotional disorders and psychosis are not as distinct as classification systems imply (Freeman and Garety, 2003), suspiciousness, like negative affect, may be another factor common to these disorders. In light of Foulds and Bedford’s (1975) model and the other models cited above, it is possible that suspiciousness is a dimensional phenomenon common to both anxiety and depression that exacerbates the symptoms of these disorders. If so, one may be able to target suspiciousness in treatment in order to reduce the severity of depression, anxiety or psychosis.

An association between suspiciousness and anxiety and depression is not unexpected, considering their common impairment in processing emotion information (e.g., Bentall and Kaney, 1989; Gur et al., 1992; Green and Phillips, 2004). However, anxiety appears more related to suspiciousness than to depression, given similar misperceptions and attributional styles. For example, both paranoia and anxiety involve a tendency to misinterpret threat which can lead to emotional arousal. However, the emotional reaction to the panic symptoms may be vague or inaccurate, leading to inaccurate judgments and delusional or peculiar beliefs (e.g., suspiciousness) about the arousal (Maher, 1974, 1988; Clark, 1986; Boden and Berenbaum, 2007). This process is supported by the fact that suspiciousness is negatively associated with clarity of emotions (e.g., the ability to identify an experienced emotion; Berenbaum et al., 2006) and positively associated with boredom proneness, which in turn is associated with hyperfocus on one’s feelings (von Gemmingen et al., 2003). In addition to a lack of clarity regarding emotions, paranoid individuals tend to blame external rather than internal sources for negative events (for a review, see Kinderman and Bentall, 1998), specifically the actions of other people (Fear et al., 1996; Kinderman and Bentall, 1997, 2000). Thus, suspiciousness may be due in part to an inability to accurately identify emotions associated with arousal in combination with an external attribution bias.

There also seems to be a cognitive component of suspiciousness that may overlap with anxiety disorders characterized by worry. For example, both suspiciousness and anxiety due to worry involve anticipation of danger (Freeman and Garety, 2003). Anxiety also contributes to the strength of suspiciousness or paranoia (i.e., conviction of persecutory delusions, Garety et al., 2005) and is a predictor of paranoia in a college student sample (Tone et al., 2011). In addition, cognitive-behavioral therapy for anxiety disorders can reduce paranoid beliefs (Key et al., 2003).

Depression and suspiciousness appear more distinct. Individuals with depression or suspiciousness do share a tendency toward hopelessness or powerlessness (Alloy and Clements, 1998) but may have different attributional styles. Paranoia is sometimes associated with attributing positive events to internal sources (e.g., Zigler and Glick, 1988; Chadwick and Trower, 1997) and attributing negative events to external sources (Fear et al., 1996; Kinderman and Bentall, 1997; Fornells-Ambrojo and Garety, 2009), whereas individuals who are depressed make negative internal attributions, in which they blame themselves for negative events (Wall and Hayes, 2000). Consequently, the association between suspiciousness and depression appears to be weaker, more indirect, or more complex than the association between suspiciousness and anxiety.

In summary, suspiciousness and anxiety share similar processes. As outlined in Grupe and Nitschke (2013), there are five processes common to anxiety disorders. It is likely that suspiciousness and anxiety associated with worry [e.g., generalized anxiety disorder (GAD)] are both associated with two of these processes: the tendency to inflate the cost and probability of threat and behavioral and cognitive avoidance. Suspiciousness and anxiety associated with arousal likely share hypervigilance under uncertainty of threat and heightened reactivity to threat. In combination, suspiciousness is likely a consequence of misinterpreting threat which leads to arousal. The inability to identify the source of these emotions in response to arousal in combination with an external attribution bias leads to apprehension and further misinterpretation of threat (anticipation of danger regarding other people). Thus, individuals who have tendency toward suspiciousness likely alternate between anxiety types (arousal vs. apprehension).

It is well-established that anxiety and depression are each associated with deficits in emotion processing (e.g., Gur et al., 1992; Heller et al., 1997; Surguladze et al., 2004; Engels et al., 2007, 2010), but suspiciousness has also been associated with deficits in emotion processing. Impaired fear processing (e.g., rating neutral antecedents of events as fear-provoking) was correlated with suspiciousness in patients with schizophrenia (Trémeau et al., 2009). This relationship is similar to the tendency of individuals with anxiety to anticipate danger in situations that others perceive as harmless. Another study indicated that individuals with persecutory delusions and a subclinical group with high paranoia scores had poorer emotion perception than did moderate and low subclinical paranoia groups. In addition, the high subclinical group showed more interference from words with paranoid content on an emotion-word Stroop task (Combs et al., 2006; see also Bentall and Kaney, 1989). This reaction to negative words has also been observed in numerous studies of anxiety (e.g., Mathews and MacLeod, 1985; Fox, 1993; Egloff and Hock, 2003). Given the clinical and conceptual overlap between suspiciousness and anxiety, it is important to determine the degree to which these two symptom dimensions reflect similar processes. In addition, given that dysfunctional emotional information processing contributes to psychopathology in general (e.g., Freeman and Garety, 2003), to the maintenance of anxiety and depression (e.g., Turk et al., 2005) and has been associated with paranoia (see above), understanding the effects of suspiciousness on emotion processing may clarify the mechanisms involved in anxiety and depression onset and maintenance as well as point to avenues for more effective intervention.

The emotion-word Stroop task is useful for investigating the role of suspiciousness in emotion processing in anxiety and depression. Behavioral interference from threat-related words has been demonstrated in this task (for review, see Williams et al., 1996) in anxiety (e.g., Mathews and MacLeod, 1985; Fox, 1993; Egloff and Hock, 2003), schizophrenia-spectrum disorders (e.g., Bentall and Kaney, 1989; Combs and Penn, 2004; Mohanty et al., 2005), and depression (Williams et al., 1996; Lim and Kim, 2005). Indices of neural activity have provided valuable information about stages of processing during this task. For example, N200 and P200 components of the event-related brain potential (ERP) have been associated with early attention to emotional stimuli in this task (e.g., Pérez-Edgar and Fox, 2003; Thomas et al., 2007; Sass et al., 2010). In other tasks, P200 is sensitive to threat perception (Carretié et al., 2001a, b; Correll et al., 2006) and N200 to cognitive control or response inhibition (Correll et al., 2006). Later processing can be measured by P300, a component that can track task difficulty and is generally interpreted as an index of the cognitive resources allocated to a task (Donchin and Coles, 1988; Yee and Miller, 1994). Negative stimuli on the emotion-word Stroop task have been associated with larger P300 (Li et al., 2007; Thomas et al., 2007), interpreted as allocation of additional resources for categorization of stimuli. Metzger and Orr (1997) reported a trend for later P300 latency to trauma-related words in patients with post-traumatic stress disorder (PTSD), indicating delayed or prolonged evaluation of such words. In combination, ES ERP studies suggest that emotion words are associated with enhanced early perception and increased allocation of resources.

Given the association between suspiciousness and anxiety, neural activity associated with suspiciousness may co-occur with and possibly influence the time course and lateralization of neural activity associated with anxiety. As psychophysiological research has supported psychometric distinctions between two dimensions of anxiety (e.g., Nitschke et al., 2001), anxious apprehension (worry, a major component of GAD) and anxious arousal (fear or somatic anxiety, a component of panic disorder and phobias), lateralization and time course of activity could be affected by the relationship of suspiciousness to each of these dimensions. Anxious apprehension or worry is associated with more left than right prefrontal activity (Heller et al., 1997; Engels et al., 2007, 2010; Mathersul et al., 2008), and there is mixed evidence for an association between apprehension and enhanced early sensory processing of emotional stimuli (e.g., Drake et al., 1991; Turan et al., 2002; Li et al., 2007; Sass et al., 2010). In contrast, anxious arousal is associated with more right than left prefrontal activity (Nitschke et al., 1999; Mathersul et al., 2008), increased right-posterior activity (e.g., Heller and Nitschke, 1998; Engels et al., 2007, 2010), and enhanced early processing (larger amplitude and shorter latency of P200; Yee and Miller, 1988; Hanatani et al., 2005; Pauli et al., 2005).

Overlapping characteristics of anxiety and suspiciousness may be associated with similar patterns of regional activity. For instance, anxious arousal and suspiciousness are both associated with arousal and consequent vigilance to threat. Thus, suspiciousness and anxious arousal combined could exaggerate vigilance characteristics, leading to increased ERP activity recorded over right-posterior cortex. In contrast, anxious apprehension and suspiciousness share verbal iterative processes (such as rumination or worry that engage areas associated with verbal processing) which involve extended processing of stimuli. Suspiciousness could combine with characteristics of anxious apprehension to augment activity manifested at left-frontal sites. Alternatively, these shared aspects associated with vigilance and verbal iterative processes could suppress the effects of each other, leading to reduced amplitude of ERP components at right-posterior and left-frontal sites.

In addition to overlap with anxiety, suspiciousness has distinct characteristics. Suspiciousness and anxious apprehension share verbal iterative processes, but suspiciousness involves concerns specifically about harm intended by others (Kinderman and Bentall, 1998), rather than excessive worry across a number of life domains (American Psychiatric Association [APA], 2000). Similarly, suspiciousness and anxious arousal are both associated with sympathetic nervous system arousal, but suspiciousness is associated with such arousal due to vigilance to external threat (e.g., people are out to get me), rather than arousal due to specific stimuli which could be external or internal (e.g., spiders, interoceptive cues). These distinct characteristics may be reflected in distinct patterns of brain activity. There is some evidence that suspiciousness is associated with activity in right temporal brain regions that have also been associated with vigilance (Robertson and Garavan, 2004). Enhanced N200 was observed over right temporal-parietal cortex during an auditory oddball task (Sumich et al., 2014), suggesting increased early attentive processes, which is consistent with a tendency toward vigilance. Fractional anisotropy in the right uncinate fasiculus, a white-matter tract that connects the temporal and frontal lobes, was correlated with suspiciousness (Nakamura et al., 2005). As anxious arousal is also associated with activity in a similar right-posterior region, it is possible that the association of suspiciousness with this area is due to overlap with anxiety.

The present study sought to identify the shared and distinct effects of trait suspiciousness and anxiety on processing of emotional information by measuring both behavioral interference and ERPs. It was hypothesized that suspiciousness would be related to early attention to all stimuli, reflected in right-temporal activity, consistent with vigilance. Second, suspiciousness would interact with anxiety dimensions to affect vigilance and verbal iterative processes, reflected in activity over right posterior and left frontal regions. P200 and N200 would index early attentive processes, and P300 would index allocation of resources to process stimuli. Depression was assessed in order to demonstrate that predicted associations with anxiety were not due to general psychopathology and because some neuropsychological, fMRI, and ERP findings for anxiety have emerged only after partialling out depression (e.g., Keller et al., 2000; Herrington et al., 2010; Sass et al., 2010). Depression was not expected to interact with suspiciousness to predict ERP measures.

## MATERIALS AND METHODS

Much of the methods section, including stimuli and experimental design, EEG recording procedure, and data reduction and analysis procedures overlap with Fisher et al. (2010) and to some extent with Sass et al. (2010, 2014) and Stewart et al. (2010).

### PARTICIPANTS

Over 1000 participants in undergraduate psychology classes filled out the Penn State Worry Questionnaire (PSWQ; Meyer et al., 1990; Molina and Borkovec, 1994) and the Anxious Arousal and Anhedonic Depression scales of the Mood and Anxiety Symptom Questionnaire (MASQ; Watson et al., 1995a, b). Five groups were recruited for a larger fMRI and EEG study based on combinations of scores on three scales: the PSWQ, the MASQ Anxious Arousal scale, and an eight-item subscale of the MASQ Anhedonic Depression scale that emphasizes depressed mood rather than low positive affect (Nitschke et al., 2001). Individuals who had scores at the 80th percentile or higher on one scale and at the 50th percentile or lower on the other two scales were recruited for three pure high-scoring groups: high anxious apprehension only (*n* = 14), high anxious arousal only (*n* = 14), or high depression only (*n* = 15). A fourth group had scores at the 80th percentile or higher on all three scales (*n* = 18), and controls had scores at the 50th percentile or lower on all three questionnaires (*n* = 27). Group criteria were for recruitment purposes only; present analyses were conducted across all participants to investigate dimensional relationships between suspiciousness, anxiety and depression^1^. All participants were right-handed, native speakers of English with self-reported normal color vision. Participants were given a laboratory tour, informed of the procedures of the study, and screened for claustrophobia or contraindications for MRI participation. The study was approved by the University of Illinois Urbana-Champaign IRB. All participants gave their informed consent prior to their inclusion in the study.

Present analyses are based on the 88 paid participants (55% female and 84% Caucasian) from which both EEG data and Suspiciousness scores from the Schizotypal Personality Questionnaire (SPQ; Raine, 1991) were obtained^2^. Participants were 18–34 years old (mean = 19.0, SD = 1.8), medically healthy by self-report, and right-handed as determined by the Edinburgh Handedness Inventory (Oldfield, 1971). Participants completed a recruitment session, a Structured Clinical Interview for DSM-IV Axis I Disorders (SCID; First et al., 1997), an fMRI session, and an EEG session. Participants completed the emotion- and color-word Stroop tasks during fMRI data and EEG. The order of presentation of the two tasks within a session was counterbalanced across subjects, as was the order of the fMRI and EEG sessions, with the SCID session in-between for most subjects. Only data from the emotion-word Stroop task during the EEG session were considered for the present report.

### MEASURES

During the recruitment session, participants were administered the SPQ and re-administered the MASQ and PSWQ. Analyses are based on these scores, since they were obtained closer in time to the EEG measurements. The test–retest reliabilities were: PSWQ, *r*(81) = 0.91, *p* < 0.001; MASQ Anxious Arousal, *r*(84) = 0.71, *p* < 0.001; and MASQ Anhedonic Depression eight-item subscale, *r*(84) = 0.64, *p* < 0.001. Data from the PSWQ was missing for four participants, so analyses using the PSWQ are based on 84 participants.

Suspiciousness scores were obtained from the eight-item true–false SPQ subscale. Examples of these items are: “I am sure I am being talked about behind my back;” “Do you sometimes get concerned that friends or coworkers are not really loyal or trustworthy?;” and “Do you often pick up hidden threats or put-downs from what people say or do?”

### TASK

Word presentation and response recording were controlled by STIM software (James Long Company, Caroga Lake, NY, USA). The present task was implemented as blocks of positive or negative emotion words alternating with blocks of neutral words, a design that has been effective (Compton et al., 2000; Bar-Haim et al., 2007). Participants received 256 trials in 16 blocks (four positive, eight neutral, four negative) of 16 trials. A trial began with the presentation of a word for 1500 ms, followed by a fixation cross for 275–725 ms (onset to onset ITI 2000 ± 225 ms). Each trial consisted of one word presented in one of four colors (red, yellow, green, blue) on a black background, with each color occurring equally often within word type (positive, neutral, negative). Each participant received one of eight orders designed to minimize stimulus order effects. In four of the eight presentation orders, the first and third blocks were neutral words, with positive and negative blocks second or fourth and valence order counterbalanced across participants. The remaining four presentation orders complemented these, with the first and third blocks being either positive or negative words and the second and fourth blocks being neutral words.

Emotional and neutral words preceded each other equally often, and no word was repeated within an experimental session. Within a block, each color appeared four times, and trials were pseudorandomized such that no more than two trials featuring the same color appeared in a row. After every fourth block, there was a brief rest period. In addition to the 16 word blocks, there were four fixation-only blocks – one at the beginning, one at the end, and two in the middle of the session. In the fixation condition, instead of a word, a brighter fixation cross was presented for 1500 ms.

The 256 word stimuli were selected from the Affective Norms for English Words (ANEW) set (Bradley and Lang, 1999). Sixty-four were positive (e.g., birthday, ecstasy, laughter), 64 were negative (e.g., suicide, war, victim), and two sets of 64 were neutral (e.g., hydrant, moment, carpet). The words were selected on the basis of established norms for valence, arousal, and frequency of usage in the English language (Toglia and Battig, 1978; Bradley and Lang, 1999) and ranged from three to eight letters in length. Words were presented in capital letters using Tahoma 72-point font at a distance of 1.35 m from the participant’s eyes, for a vertical span of 1.2° and a horizontal span of 3.2° to 9.1°. Instructions were read verbatim by experimenters to assure that participants understood task requirements. The participant performed 32 practice trials before the actual tasks began. No participants failed to understand the task instructions or the mapping between colors and buttons after completing practice trials. Participants responded with the middle and index fingers of each hand using a four-button response box.

### ELECTROPHYSIOLOGICAL RECORDING

Subjects were seated in a comfortable chair in a quiet room connected to the adjacent equipment room by intercom. EEG was recorded with a custom-designed Falk Minow (Munich, Germany) 64-channel cap with Ag/AgCl EEG electrodes spaced equidistantly. The left mastoid served as the reference during recording (Miller et al., 1991; Keil et al., 2014). By placing electrodes above and below each eye and near the outer canthus of each eye, vertical and horizontal EOG were recorded. Electrode impedances were maintained below 20 kohms. This impedance threshold was appropriate because the amplifier (James Long Company, Caroga Lake, NY, USA) had a high input impedance (10 GΩ Keil et al., 2014). Half-power amplifier bandpass was 0.1–100 Hz, with digitization at 250 Hz.

### DATA REDUCTION

Artifacts were removed and eye movement artifact corrected with Brain Electrical Source Analysis (BESA v. 5.1.8) software (Berg and Scherg, 1994). Trials were rejected if reaction time (RT) was not between 200 and 1000 ms, as responses less than 200 ms would be made too soon after stimulus onset and thus would not be credible, and responses greater than 1000 ms would likely reflect trials in which the participant was not engaged in the task. Mean RT across all trials and participants was 633 ms, SD 97 ms. For each subject, all trials for each emotion word type were averaged, since the error rate was low (4.5%, SD 3.9%), and the phenomena of interest were not expected to vary according to error rates. The electrode configuration was transformed to BESA’s standard 81-channel virtual montage placed according to the 10–10 system (Perrin et al., 1989) to facilitate comparison with literature that reports data from conventional electrode sites. An average reference was computed for each time point as the mean voltage over the 81 virtual electrodes. Data were exported from BESA and baseline-adjusted by subtracting the average amplitude for the 200 ms before stimulus onset. Waveform averages were smoothed using a 101-weight, 0.1–10 Hz (half-amplitude) FIR digital filter (Cook and Miller, 1992; Nitschke et al., 1998; Edgar et al., 2005). Amplitude and latency scores were obtained for ERP components at each of the 81 electrodes.

**FIGURE 1:**
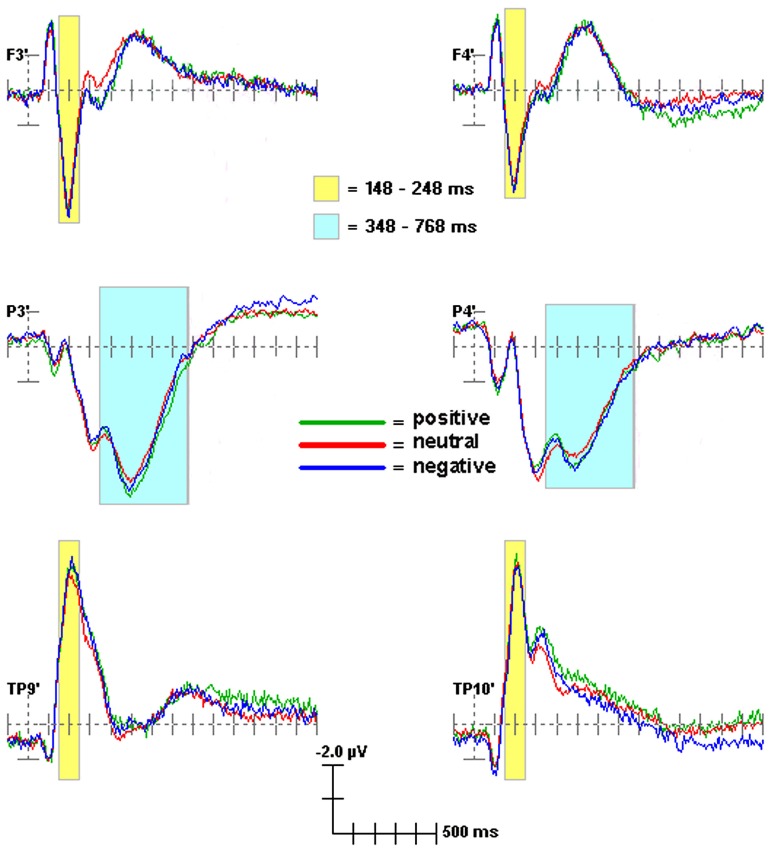
**ERP scoring windows illustrated for representative channels**. All channels relative to an average reference. Waveforms from 100 ms prior to stimulus onset to 1400 ms after stimulus onset. Each tick mark on the x-axis represents 100 ms. The apostrophes after the channel names indicate that the channel locations were digitized.

**FIGURE 2:**
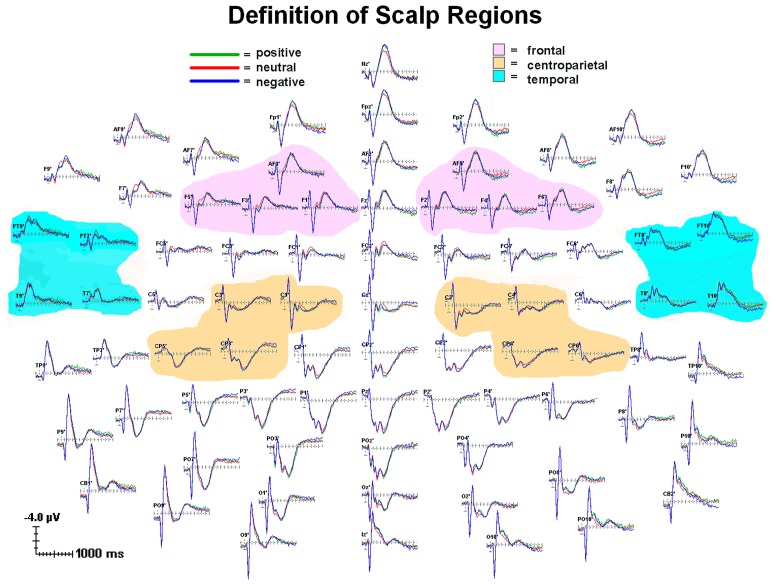
**Grand average (*n* = 88) for each emotion condition showing scalp topography for individual channels and regional groupings of channels**. All channels relative to an average reference. Waveforms from 100 ms prior to stimulus onset to 1400 ms after stimulus onset. Each tick mark on the x-axis represents 100 ms. The apostrophes after the channel names indicates that the channel locations were digitized.

Scoring windows for ERP components were chosen by examining the data and consulting previous Stroop and other ERP studies. **Figure 1** illustrates temporal scoring windows, and **Figure 2** illustrates grouping of adjacent sites by region for analysis purposes. For each participant, peak amplitude was calculated within the following latency windows and regions: P200 (148–248 ms; frontal), N200 (148–248 ms; temporal), and P300 (348–768 ms; centroparietal). P200 and N200 were scored as the peak amplitude in the same 148–248 ms latency window. These two peaks are readily apparent in **Figures 1** and **2**. Whether they represent distinct phenomena, or opposite poles of the same dipole, is not as clear. Of the three traditional criteria for defining an ERP component, they share latency, and their topographies are sufficiently complementary to be compatible with a single dipole per hemisphere. On the third criterion, however, they diverge consistently, showing distinct relationships to experimental manipulation^3^. Thus, they were analyzed separately (see **Table 1**).

**Table 1:** Mean (SD) ERP scores for negative stimuli in each region.

	P200 peak amplitude and latency	N200 peak amplitude and latency	P300 peak amplitude and latency
Left frontal	3.2 (2.3) μV		
	200 (19) ms		
Right frontal	2.9 (2.2) μV		
	200 (22) ms		
Left temporal		-2.5 (1.3) μV	
		217 (34) ms	
Right temporal		-2.4 (1.7) μV	
		V 216 (27) ms	
Left centroparietal			2.8 (1.9) μV
			530 (91) ms
Right centroparietal			2.8 (1.6) μV
			540 (105) ms

Groups of four adjacent electrodes were selected to create three composite region scores in each hemisphere to obtain a stable measure of activity. For each ERP component, a regional score was calculated for each hemisphere by averaging the scores of the individual electrodes over the region, thus creating six scores (frontal: left: AF3, F1, F3, F5; right: AF4, F2, F4, F6; Centroparietal: left: C1, C3, CP3, CP5; right: C2, C4, CP4, CP6; Temporal: left: FT9, FT7, T9, T7; right: FT8, FT10, T8, T10; see **Figure 2**). Frontal sites were chosen for P200, as maximal effects were observed there in this dataset and in prior literature (Luck, 2005; Pauli et al., 2005). Temporal sites were chosen for N200 as maximal effects associated with suspiciousness were observed there in prior literature (Sumich et al., 2014). Centroparietal sites were chosen for P300 (Luck, 2005). Scores from each hemisphere were obtained separately in order to establish whether the results were specific to sites over the predicted hemisphere^4^. Thus, there were three ERP measures (P200 over frontal; N200 over temporal; P300 over centroparietal) in each hemisphere.

### DATA ANALYSIS

A number of ERP studies of emotion have observed P300 amplitude modulation by emotional stimuli, with positive and negative stimuli prompting large responses (e.g., Schupp et al., 1997, 2003; Herbert et al., 2006). Accordingly, P300 was examined to determine whether the task manipulation was effective. To determine whether the task manipulation was successful and to facilitate comparison with prior literature, a 2 × 3 (hemisphere × emotion) repeated-measures MANOVA including linear and quadratic trends was conducted for P300 scores.

Because the primary constructs and measures of interest were continuous, regressions were used to test whether anxiety, depression and suspiciousness scores predicted ERP component scores at hypothesized scalp regions. In order to minimize the number of regressions calculated and to simplify interpretation, multivariate linear regressions were conducted so that multiple dependent variables could be simultaneously entered in the models, instead of predicting each ERP in each condition over each hemisphere. This analysis strategy is more conservative and is less likely to uncover spurious effects than running separate analyses. Two sets of hierarchical regressions were conducted to investigate whether suspiciousness has either an additive or interactive relationship with anxiety (or depression), and how this relationship affects emotional information processing. The first set of regressions determined whether suspiciousness accounted for additional variance when added last to regression models in which anxious apprehension, anxious arousal, and anhedonic depression scores were already entered simultaneously as predictors of ERP amplitude scores for emotion (positive, neutral, and negative) stimuli over right and left hemispheres. Thus, there were four predictors and six dependent variables in each regression model (Model 1).

The second set of regressions investigated whether anxiety or depression interacted with suspiciousness or provided additive effects. Either an anxious apprehension (Model 2), anxious arousal (Model 3) or anhedonic depression (Model 4) score was entered first, suspiciousness was entered second, and an interaction term (product of two centered variables, per Cohen et al., 2003) was added third to predict the six ERP amplitude scores for each ERP component. All statistical analyses were conducted using SPSS v. 12 for Windows.

## RESULTS

### PSYCHOPATHOLOGY MEASURES AND BEHAVIORAL PERFORMANCE

Mean RT did not differ as a function of emotion (positive: 634 ms, SD 95 ms; neutral: 631 ms, SD 96 ms; negative: 633 ms, SD 101 ms)^5^. Although correlations with RT were somewhat higher for suspiciousness than for anxiety and depression, only one was significant (see **Table 2**). Thus, self-reported symptoms were generally not related to overt performance, avoiding some potential interpretive confounds such as individuals scoring high in depression being less motivated for task engagement.

**Table 2:** Correlations among suspiciousness, anxiety, and depression measures and behavioral performance on the emotion-word Stroop task.

	Anxious apprehension	Anxious arousal	Anhedonic depression	Positive-word RT	Neutral-word RT	Negative-word RT
Suspiciousness	0.46**	0.41**	0.42**	–0.22*	–0.12	–0.18
Anxious apprehension		0.35**	0.43**	–0.07	–0.11	–0.08
Anxious arousal			0.42**	0.01	0.06	0.01
Anhedonic depression				–0.02	0.05	0.02
Positive-word RT					0.93**	0.91**
Neutral-word RT						0.92**

Note. For n = 88, two-tailed Pearson correlation. *p ≤ 0.05, **p ≤ 0.01.

**Table 2** shows that zero-order correlations among self-reported anxiety, depression and suspiciousness scores were positive and reliable. The relationship of suspiciousness to anxious apprehension, anxious arousal, and anhedonic depression was further examined in a hierarchical regression. The full three-predictor model accounted for 33% of the variance in suspiciousness, *F*(3,79) = 13.05, *p* < 0.001. Each predictor contributed unique variance when added last (anxious apprehension: Δ*R*^2^ = 0.05, *p* = 0.02; anxious arousal: Δ*R*^2^ = 0.05, *p* = 0.02; depression: Δ*R*^2^ = 0.04, *p* = 0.03), indicating that depression and the two dimensions of anxiety have distinct as well as overlapping relationships to suspiciousness.

### ERP ANALYSES

#### Manipulation check

To determine whether the emotion-word Stroop task manipulation was successful and to facilitate comparison with prior literature, a 2 × 3 (hemisphere × emotion) repeated-measures MANOVA including linear and quadratic trends was conducted to predict P300. As expected, there was a main effect of Emotion, *F*(2,86) = 6.04, *p* = 0.004. A quadratic effect confirmed that P300 to positive and negative emotion stimuli was larger than P300 to neutral stimuli, *F*(1,87) = 12.17, *p* = 0.001.

#### P200

***Model 1.*** In order to determine whether suspiciousness, in the context of anxiety and depression, had an additive effect on initial stimulus perception, it was entered in a multivariate model with the three other psychopathology measures. None of the four predictors was significant (*p’*s = 0.16–0.93).

**FIGURE 3:**
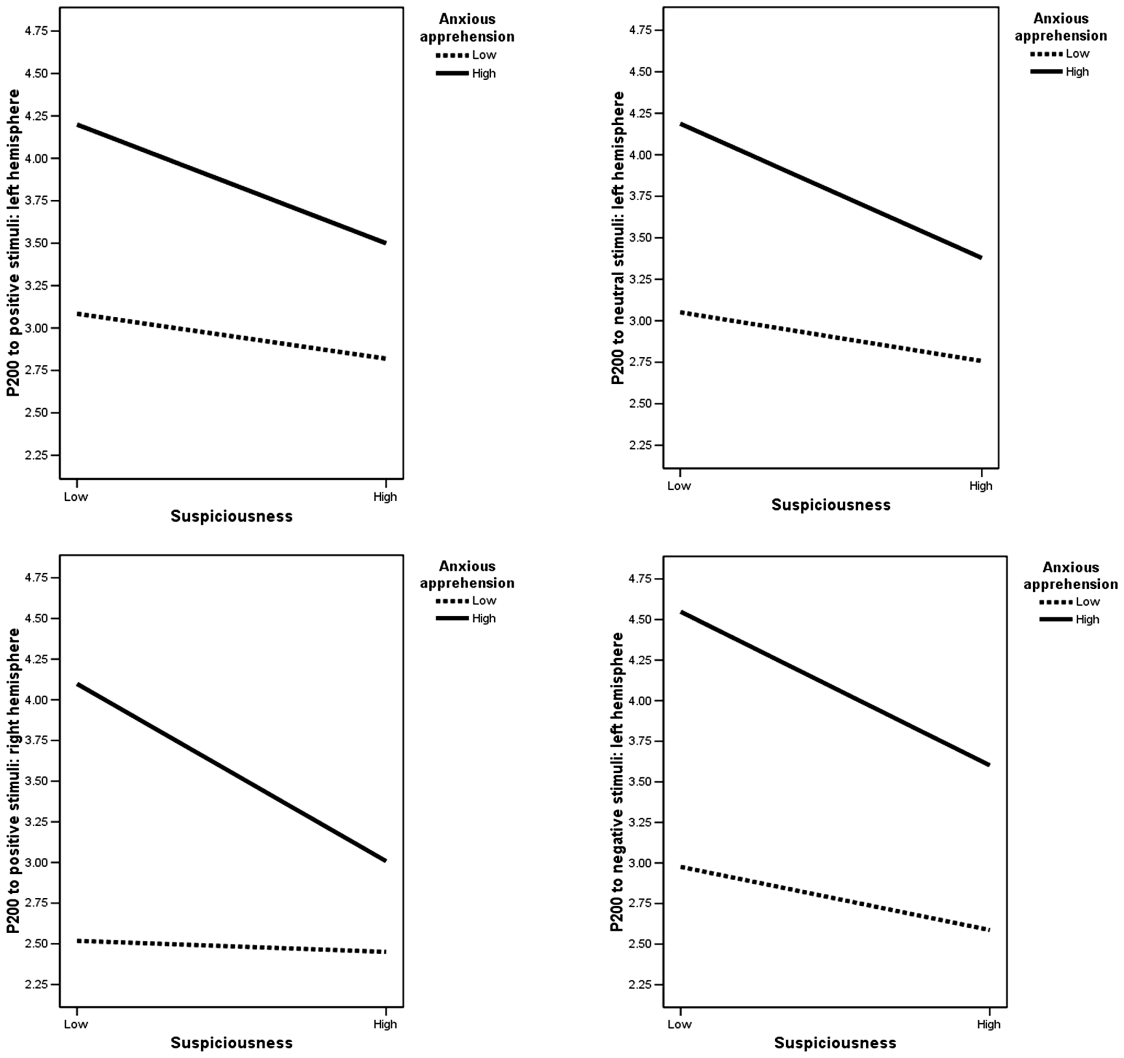
**Anxious apprehension × suspiciousness interactions**. *Y* values are P200 amplitude scores to stimuli over the specified hemisphere. Low and high labels represent suspiciousness and anxious apprehension scores divided according to a median split.

***Model 2.*** Neither anxious apprehension (*p* = 0.51) nor suspiciousness (*p* = 0.62) predicted P200, but the interaction of suspiciousness and anxious apprehension did (*F*(6,75) = 2.38, *p* = 0.04). Univariate multiple linear regressions indicated that the interaction predicted P200 to positive stimuli over the right hemisphere (*B* = –0.54, Δ*R*^2^ = 0.05, *p* = 0.05), and predicted P200 to each condition (including neutral) over the left hemisphere, though the positive condition was only marginally significant (positive: *B* = –0.50, Δ*R*^2^ = 0.04, *p* = 0.06; neutral: *B* = –0.56, Δ*R*^2^ = 0.05, *p* = 0.03; negative: *B* = –0.74, Δ*R*^2^ = 0.07, *p* = 0.01). As illustrated in **Figure 3**, individuals with high scores on both suspiciousness and anxious apprehension had a reduced amplitude compared to those with a combination of low suspiciousness and high anxious apprehension. The same was true for high suspiciousness/low anxious apprehension compared to individuals with low scores on both measures.

Neither Model 3 nor Model 4 accounted for variance in P200.

#### N200

***Model 1.*** With all four predictors (anxious apprehension, anxious arousal, anhedonic depression, and suspiciousness) in the multivariate linear regression model, suspiciousness was the only significant predictor (*F*(6,73) = 2.27, *p* = 0.05). This effect was examined further with univariate multiple regressions. When added last to the model, suspiciousness predicted N200 to positive (*B* = –0.19, Δ*R*^2^ = 0.06, *p* = 0.03) and to negative (*B* = –0.27, Δ*R*^2^ = 0.10, *p* = 0.002) stimuli over the right hemisphere. It accounted for variance at a trend level for P200 to neutral stimuli over the right hemisphere (*B* = –0.15, Δ*R*^2^ = 0.03, *p* = 0.10). Though not significant in the multivariate model, anxious arousal (*B* = 0.05, Δ*R*^2^ = 0.05, *p* = 0.02) and suspiciousness were both predictors of N200 in response to negative stimuli over the right hemisphere, though in opposite directions.

***Model 2.*** Model 2 did not account for significant variance in N200.

***Model 3.*** Suspiciousness was a significant predictor (*F*(6,78) = 4.41, *p* = 0.001) in the model with anxious arousal and the interaction between anxious arousal and suspiciousness. Consistent with the Model 1 regression, univariate regressions for each of the emotion conditions indicated that suspiciousness predicted N200 over the right hemisphere to positive (*B* = –0.18, Δ*R*^2^ = 0.05, *p* = 0.02), neutral (*B* = –0.19, Δ*R*^2^ = 0.07, *p* = 0.01) and negative (*B* = –0.34, Δ*R*^2^ = 0.19, *p* < 0.001) stimuli.

***Model 4.*** Suspiciousness predicted N200 at a trend level (*F*(6,78) = 2.03, *p* = 0.07). Anhedonic depression and the interaction between anhedonic depression and suspiciousness were not significant predictors of N200 (anhedonic depression *p* = 0.53; interaction *p* = 0.86).

#### P300

***Model 1.*** Anxious arousal predicted P300 at a trend level (*F*(6,73) = 2.16, *p* = 0.06). None of the other predictors was significant.

***Model 3.*** When anxious arousal, suspiciousness, and their interaction were predictors, only anxious arousal was significant at a trend level (*F*(6,78) = 2.00, *p* = 0.08). Univariate regressions with the same predictors indicated that anxious arousal predicted P300 to positive (*B* = 0.05, Δ*R*^2^ = 0.04, *p* = 0.04) and negative stimuli (*B* = 0.06, Δ*R*^2^ = 0.08, *p* = 0.01) over the right hemisphere. However, when the order of predictors was reversed and suspiciousness was entered first, it was a significant predictor, but only for P300 to negative stimuli over the right hemisphere (*B* = 0.15, Δ*R*^2^ = 0.04, *p* = 0.05). Once anxious arousal was entered in the model, suspiciousness was no longer significant. These regressions indicate that shared characteristics of suspiciousness and anxious arousal (e.g., vigilance) predict P300 to negative stimuli over the right hemisphere.

Models 2 and 4 were not significant.

## DISCUSSION

The present study investigated whether suspiciousness affects processing of emotional information in a unique manner, or whether it overlaps with anxiety to affect processing. Teasing apart the relationship between anxiety and suspiciousness would foster improved definition, classification, and treatment of psychological disorders. Behavioral and ERP indices were used. The larger P300 for positive and negative than neutral stimuli indicated a successful emotional arousal manipulation. Study hypotheses specified that suspiciousness alone would be related to early attention to emotion information, reflected by right-temporal activity, and that suspiciousness would interact with anxiety to affect vigilance and verbal iterative processes in response to emotion information, reflected in activity over right posterior and left frontal regions.

Supporting the first hypothesis, high suspiciousness was associated with enhanced right temporal N200 to all stimuli. The other psychopathology measures did not show such a relationship. This finding for suspiciousness is consistent with the limited research available that links activity of the temporal region to suspiciousness (Li et al., 2011), especially the right temporal region (Nakamura et al., 2005; Sumich et al., 2014). Associations with the right temporal lobe may be due to involvement of a right frontoparietal vigilance system that modulates arousal (Nitschke et al., 2000; Robertson and Garavan, 2004). The right ventral frontoparietal network has been implicated in attention to behaviorally relevant stimuli and has been activated during “theory of mind” cognition (involving judgments of another person’s mental state), thus requiring a combination of perceptual processes and judgment of other people’s actions (Corbetta et al., 2008). The temporal N200 in the present study can be distinguished from a fronto-central N200 that is thought to be associated with effortful processing (e.g., Donkers and van Boxtel, 2004), and typically peaks later in time (between 200 and 500 ms; e.g., Thomas et al., 2007; Enriquez-Geppert et al., 2010). Present results suggest that suspiciousness is associated with an overactive early attentive response (manifested in N200) to any type of stimulus, regardless of its emotion content. Thus, individuals with high suspiciousness scores likely judged all stimuli to be behaviorally relevant. Though this study investigated suspiciousness in a non-clinical sample, this hyperactive response to all stimuli is consistent with reports of misattribution of salience to neutral stimuli in patients with schizophrenia (Holt et al., 2006; Heerey and Gold, 2007). Similarly, neutral and negative stimuli led to increased mesotemporal and ventral striatal activity and reduced prefrontal activity in patients with hallucinations and delusions, whereas controls showed this response to negative stimuli only (Epstein et al., 1999). Thus, it appears that suspiciousness, even at non-clinical levels in the general population, influences the perception of stimuli in the same manner as that observed in clinical populations.

The second hypothesis was that suspiciousness would combine with anxiety to affect emotion processing, specifically that the interaction of suspiciousness and anxious apprehension would affect left-frontal activity and that suspiciousness and anxious arousal would affect right-posterior activity. The interaction of suspiciousness and anxious apprehension did predict P200 (index of stimulus perception) to neutral and negative stimuli over the left hemisphere, consistent with fMRI evidence that left-hemisphere activity is associated with anxious apprehension (e.g., Engels et al., 2007, 2010). To interpret this P200 interaction, characteristics of anxious apprehension and anxious arousal that are shared with suspiciousness were used as a guide (see **Figure 3**). Given that suspiciousness and anxious apprehension presumably share verbal-iterative processing, an additive effect might have been expected. Instead, individuals with high scores on both suspiciousness and anxious apprehension had a reduced amplitude compared to those with a combination of low suspiciousness and high anxious apprehension. The same was true for high suspiciousness/low anxious apprehension compared to individuals with low scores on both measures. Thus, the presence of high levels of suspiciousness in the context of anxiety reduced P200 amplitude. It is possible that the aspect of suspiciousness associated with arousal and vigilance (involving the right hemisphere) may have reduced the degree to which verbal-iterative processing (left hemisphere processes) associated with anxious apprehension was reflected. Unexpectedly, the suspiciousness × anxious apprehension interaction also predicted P200 to positive stimuli over the *right* hemisphere in the same manner. This finding could be explained by the fact that suspiciousness is associated with responses to all types of stimuli. Therefore, instead of finding an expected association between anxious apprehension and a response to negative stimuli only, the presence of suspiciousness led to a more generalized response to all stimuli.

Contrary to hypotheses, suspiciousness did not interact with anxious arousal to predict ERP measures over the right-posterior region. Instead, anxious arousal and suspiciousness each were independent, but overlapping predictors of N200 and P300 to negative stimuli over the right hemisphere. When both were entered in a regression model, only one accounted for variance (suspiciousness for N200 over right temporal region and anxious arousal for P300 over right centroparietal region), indicating that suspiciousness and anxious arousal share overlapping characteristics that predict response to negative stimuli over the right hemisphere. The association of anxious arousal with P300 over the right centroparietal region is consistent with evidence that right-central (e.g., inferior temporal gyrus, Engels et al., 2007) and right-temporoparietal (Heller et al., 1997; Keller et al., 2000; Compton et al., 2000, 2003) areas are associated with anxious arousal and a network involved in vigilance to behaviorally relevant stimuli (Tucker and Williamson, 1984; Heller, 1990; Heller et al., 1998; Nitschke et al., 1999, 2000; Herrington et al., 2005; Corbetta et al., 2008).

Present results indicate that anxiety and suspiciousness each affects emotion processing alone, but also in combination. Suspiciousness interacts with anxious apprehension to modulate initial stimulus perception processes, manifested in P200 recorded over frontal cortex. In addition, suspiciousness and anxious arousal share overlapping characteristics that predict response to negative stimuli over the right hemisphere. Finally, suspiciousness is uniquely associated with a hyperactive early response (enhanced N200 to all stimuli). Together, these results suggest that suspiciousness is associated with hypervigilance to all incoming stimuli and interacts with anxiety to modulate early attention to emotion stimuli. As predicted, suspiciousness did not interact with anhedonic depression to predict ERP measures.

Given that suspiciousness is present in individuals with anxiety symptoms and that it is uniquely associated with early processing of incoming information (with emotion content or otherwise), suspiciousness should be assessed more frequently in individuals who present with anxiety symptoms in order to determine whether these individuals perceive threat in positive or neutral situations. Potential treatments could target reducing vigilance to perceived threat (common in anxious arousal) and minimizing verbal-iterative processes about anxiety-provoking events or thoughts (common in anxious apprehension). Depression, which often co-occurs with anxiety and suspiciousness, should be treated separately, as it appears to be a distinct construct. In summary, these results identify how characteristics of suspiciousness, both unique and those that overlap with two anxiety dimensions, affect processing of emotion information. The characterization of these symptom dimensions provides additional support for the recent emphasis (e.g., NIMH Research Domain Criteria (RDoC) project) on using dimensions to classify psychopathology. In addition, these results extend prior findings to a non-clinical population and suggest ways to refine treatments for individuals with clinically significant levels of suspiciousness and anxiety.

## Conflict of Interest Statement

The authors declare that the research was conducted in the absence of any commercial or financial relationships that could be construed as a potential conflict of interest.

## Footnotes

1 As discussed in Fisher et al. (2007), it can be problematic to use correlation or regression analysis with an extreme-groups design. However, the present study included subjects with scores that encompassed most of the distribution. Simulations with randomly generated data indicated that results were not substantially affected for the distribution used here versus the full simulated dataset (Fisher and Miller, 2005).

2 The selection method did not provide a sample of suspiciousness representative of the general population but instead allowed controlled representation of specific types and amounts of anxiety and depression. Of the present sample, 38 of the 88 participants were included in Sass et al. (2010), which analyzed data from anxious apprehension, anxious arousal, and control groups and did not address suspiciousness. The present sample also included a combined anxiety and depression group. In addition, all of the present sample was included in Fisher et al. (2010), which investigated the role of perceived emotional intelligence, anxiety, and depression in processing emotion stimuli and did not address suspiciousness.

3 To ensure that the components observed during the same time window over different regions of the scalp were not manifestations of the same dipole, time-point-by-time-point correlations were done between an exemplar electrode in the frontal region (AF3) and one in the posterior region in the opposite hemisphere (PO4). Correlations were generally <0.1 for P200/N200 indicating that these components are independent.

4 Laterality effects were calculated for an earlier set of analyses (Fisher, 2006). However, they were mostly redundant with the present findings and were not included in order to simplify presentation of results.

5 Both positive and negative stimuli produced very slightly and non-significantly slower responses than did neutral stimuli. Other studies that have used the ES task in non-clinical samples have not found reliable behavioral effects but have observed changes in neural activity (e.g., Whalen et al., 1998; Compton et al., 2003). The absence of behavior effects likely reflects small sample sizes, as a large sample is needed to reveal this effect in a non-clinical sample (Koven et al., 2003).
